# Research on Filling Strategy of Pipeline Multi-Layer Welding for Compound Narrow Gap Groove

**DOI:** 10.3390/ma15175967

**Published:** 2022-08-29

**Authors:** Tie Yin, Jinpeng Wang, Hong Zhao, Lun Zhou, Zenghuan Xue, Hehe Wang

**Affiliations:** 1 College of Mechanical and Transportation Engineering, China University of Petroleum-Beijing, Beijing 102249, China; 2 China Petroleum and Natural Gas Pipeline Research Institute, Langfang 065000, China; 3 713th Research Institute of China State Shipbuilding Corporation, Zhengzhou 450015, China

**Keywords:** pipeline welding, narrow gap groove, filler layer strategy, prediction model

## Abstract

With the increase in transmission pressure and pipe diameter of long-distance oil and gas pipelines, automatic welding of the pipeline has become the mainstream welding method. The multi-layer and multi-pass welding path planning of large-diameter pipelines with typical narrow gap grooves are studied, and a welding strategy for pipeline external welding robot is proposed. By analyzing the shape of the weld bead section of the narrow gap groove and comparing the advantages and disadvantages of the equal-height method and the equal-area method, the mathematical model of the filling layer is established. Through the test and analysis in the workshop, the predicted lifting value meets the actual welding requirements. The microstructure of the weld was analyzed by SEM. The main structure of the weld was fine acicular ferrite, which could improve the mechanical properties of the welded joint. After multi-layer filling, the filling layer is flush with the edge of the groove. The establishment of this model lays a foundation for the formulation of welding process parameters for large-diameter pipes and the off-line programming of welding procedures.

## 1. Introductions

**Figure 1:**
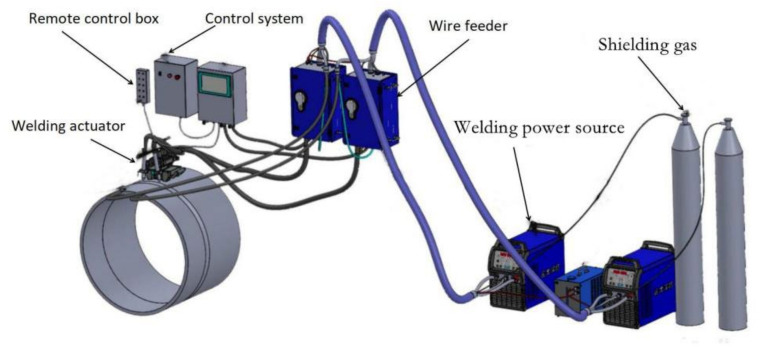
Pipeline all-position welding system.

With the constant expansion in natural gas consumption in China in recent years, the Belt and Road Initiative development policy has been built up, and the construction of oil and gas pipelines, as symbolized by the Sino-Russian gas pipeline project, has set off a pipeline construction boom. Automatic welding of pipelines has the advantages of small welding heat input, the stable mechanical joint performance of circumferential weld, good adaptability to the metallurgical composition and rolling process of steel pipe base metal, high degree of automation of welding operation, stable welding quality, and so on. Automatic welding is the most widely used form of pipeline welding now. The pipeline is all-position welded by securing the steel pipe and moving the welding robot. The pipeline welding robot moves along the circumference of the pipe [1,2,3]. As shown in Figure 1, the system includes a welding actuator, remote control box, wire feeder system, control system, welding machine and other parts. The function of the welding actuator is to drive the actuator to rotate on the circular track, and the welding torch is fixed on the actuator to follow the rotary motion [4,5,6,7,8,9].

Currently, the teaching method is used for the automatic welding of large-diameter pipes, and it is necessary to program the welding robot’s welding process parameters, motion control program, and motion trajectory program in advance using information from the welded workpiece such as pipe diameter and wall thickness. As a result, planning the filling strategy of multi-pass welding to set the filling sequence of each bead and the welding parameters of each bead or layer of bead is important to simplify the welding robot’s training task [10,11,12,13,14,15].

**Figure 2:**
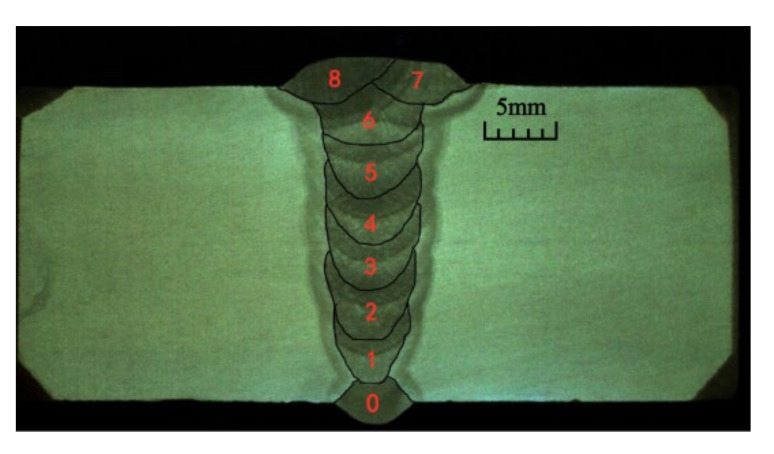
Narrow gap groove weld.

Large-diameter oil and gas pipelines usually adopt the MIG/MAG welding process. The welding method of inner welding machine root welding and outer welding machine filling cover welding is commonly used for automatic welding of large diameter pipes. The pipe mouth group is welded to the internal root of the pipeline by the welding robot first, and then the external welding completes the external filling welding and covering welding, as shown in Figure 2, where layer 0 represents the root welding layer, layer 1–6 represents the filling layer, and layer 7–8 represents the cover layer. The first layer completes the fusion with the root weld. Due to a large number of filling layers, the probability of defects is greatly increased. Ideally, it is hoped that the filler metal can be flush with the outside of the steel pipe after filling, laying a good foundation for cover welding. By studying the filling strategy of typical multilayer and multi-pass welding of narrow gap grooves and establishing the mathematical model of filling layer and the prediction model of lifting capacity, the welding process parameters can be optimized and guided, and the exploration time of the welding process parameters can be reduced.

## 2. Weld Bead Filling Strategy for the Cross-Sectional Area of Multi-Layer and Multi-Pass Welding of Pipeline

### 2.1. Basic Thought

The commonly used filling strategies of welding robots outside the pipeline with narrow gap grooves in the filling layer of pipeline weld are the equal height method and the equal area method. The equal height method makes the layer height of each weld consistent by controlling the corresponding welding parameters. Since the automatic welding of pipelines adopts all-position welding, the contour method requires frequent parameter changes during the welding process, which is difficult to achieve precise control. The equal area method makes the cross-sectional area of each weld the same by controlling the corresponding welding parameters [16,17,18,19,20].

**Figure 3:**
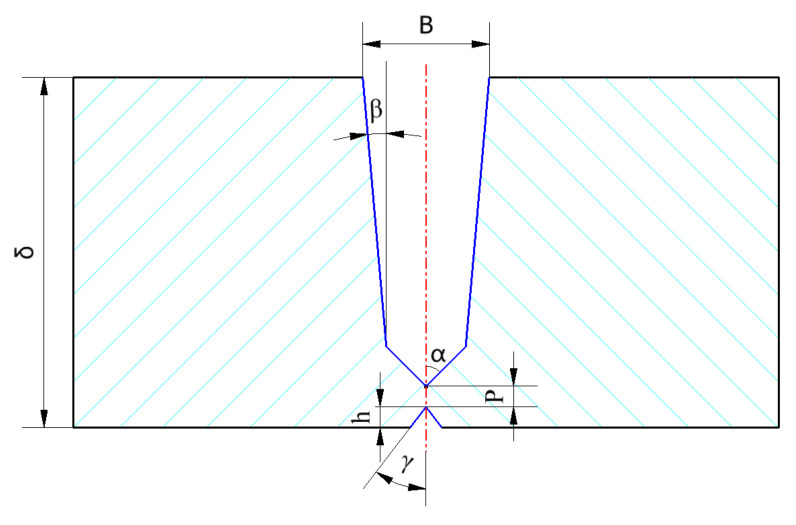
Schematic diagram of narrow gap compound groove.

The groove type shown in Figure 3 is the narrow gap compound groove type, and the joint type is the butt joint. According to the welding process requirements, the automatic welding of large-diameter oil and gas pipelines usually adopts the form of a compound groove. The corresponding parameters are as follows: groove surface angle: α = 45°, β = 5°, γ = 37.5°. The blunt edge (P) is 1.1~1.3 mm, the height *h* from the inflection point to the inner wall is 5.1 mm, the height *h* of the inner groove is 1.3 mm, and B is the maximum weld width, δ Indicates the pipe wall thickness.

**Figure 4:**
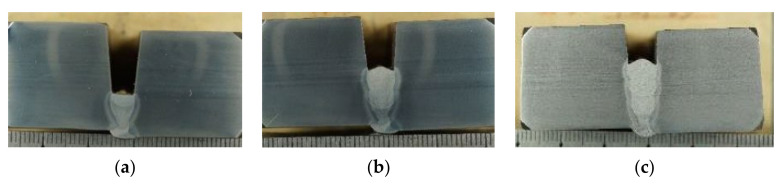
Weld pass filling layer appearance: (**a**) concave; (**b**) convex; (**c**) hump.

Only single-pass welding is required for each layer of filler layer. The welding process adopts downward welding. Good mechanical properties of the weld are obtained after extensive welding experiments. The fully automatic welding process of solid wire, the tensile strength of the weld is about 720–780 MPa, and the impact energy at −10 ℃ is 150–200 J, with excellent performance. According to the process characteristics of MAG welding, gas-shielded solid wire is used for welding. It is better to determine the lifting amount *h_i_* in the range of 2.0~3.0 mm. The actual pipeline welding layer has a certain radian in the welding process due to various reasons. Such as concave, convex, and hump [20,21,22,23,24,25], as shown in Figure 4.

**Figure 5:**
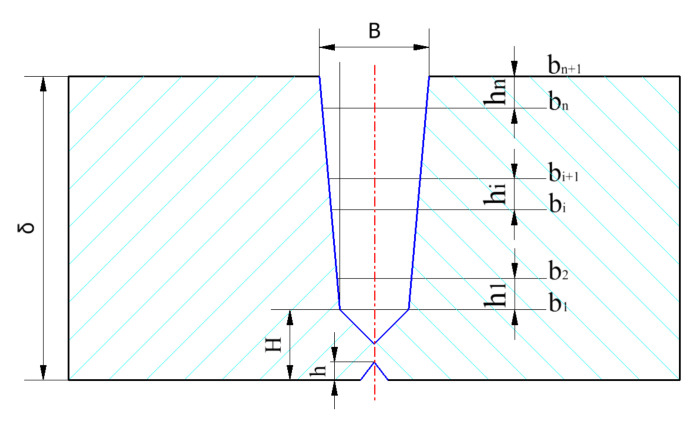
Filling diagram of pipeline external welding robot.

To facilitate the study of the filling strategy of the weld filling layer, simplified processing is carried out, and the actual filling layer is treated as an isosceles trapezoid. The schematic diagram of the filling is shown in Figure 5.

(1) Equal height method
  The equal height method is a method often used in pipeline welding in engineering. It only needs to meet the same layer height of each filling layer, that is, it needs to meet the following conditions:
  (1) $$h_{1}=h_{2}=\cdots =h_{i}=\cdots =h_{n}=\frac{\delta -H}{n}$$
  where *i =* 1, 2*,…, n, n* is the number of filling layers.
  The pipeline external welding robot’s motion planning and design just require setting the same value in the radial direction of the pipeline (i.e., the external welding robot’s lifting mechanism) every time.
(2) Equal area method
  The equal area method needs to solve the welding layer height of the filling welding layer to set the lifting amount of the welding gun of the external welding robot. The cross-sectional area of each weld layer is equal, so the total area of the weld cross-section is:
  (2) $$\sum_{i=1}^{n}S_{i}=nS_{i}=\frac{1}{2}\times \left(\delta -H\right)\times \left(b_{1}+b_{n+1}\right)$$
  The recurrence relationship of weld width of adjacent weld layers is as follows:
  (3) $$b_{i+1}=b_{i}+2\times h_{i}\times \tan\beta$$
  The relationship between the weld width of the initial weld layer and the maximum weld width of the weld is as follows:
  (4) $$b_{n+1}=b_{1}+2\times \left(\delta -H\right)\times \tan\beta$$
  where, *i =* 1, 2,*…*, *n, n* is the number of filling layers.

Because each welding layer can theoretically be divided into an isosceles trapezoid because the narrow gap compound groove is symmetrical about the weld center, the cross-sectional area of each filling layer can be calculated as:

(5) $$S_{i}=\left(b_{i}+h_{i}\tan\beta \right)\times h_{i}$$

Equation (6) can be transformed into a univariate quadratic equation about *h_i:_*

(6) $$\tan\beta \times h_{i}^{2}+b_{i}\times h_{i}-S_{i}=0$$

The layer height *h_i_* of each filling layer is obtained by the formula

(7) $$h_{i}=\frac{-b_{i}+\sqrt{b_{i}^{2}+4\times S_{i}\times \tan\beta }}{2\times \tan\beta }$$

where the value of *S_i_* is

(8) $$S_{i}=\frac{1}{2n}\times \left(\delta -H\right)\times \left(b_{1}+b_{n+1}\right)$$

Then, repeatedly calculate the values of *b_i_* and *h_i_* (*i =* 1,2,…,*n*) according to Formula (3) and Formula (7), that is, A series of values of *b_i_* and *h_i_* (*i =* 1,2,…,*n*) can be obtained from the calculation sequence of according to *b*_1_→*h*_1_→*b*_2_→*h*_2_→…→*b_i_→h_i_→…→b_n_→h_n_*, in which *h_i_* (*i =* 1,2,…,*n*) is the height of each layer of the filling layer under the equal area method, that is, the lifting amount of the lifting mechanism of the external welding robot.

It can be seen from Figure 4 that the layer height *h_i_* (*i =* 1,2,…,*n*) of the filling layer follows a strict decreasing law.

### 2.2. Application Analysis of Equal Height Method and Equal Area Method

The filling layer height of the pipeline with a diameter of 1219 mm, a wall thickness of 22 mm, and a filling layer height of 16.9 mm is calculated using the equal height and equal area methods, as shown in Table 1.

The lifting (The lifting height of the torch during welding of each layer) amount of each filling layer is managed within the range of 2.0 mm 3.0 mm, which is commonly controlled at about 2.5 mm to ensure the needs of corresponding welding stress, according to real welding experience. Usually, small-diameter pipes are controlled between 2.0~2.5 mm, and large-diameter pipes are controlled between 2.5~3.0 mm to ensure the actual welding application needs. The filling layer height of the contour method is the same, but the contour method does not consider the different weld widths *b_i_* of each weld bead of the filling layer, resulting in a large difference in the welding cross-sectional area; due to the same theoretical filling area of each filling layer, the equal area method cannot ensure that the height of each weld bead is concentrated in the range of 2.0~3.0 mm. Table 1 shows that the first and second layer heights are both more than 3 mm, making it impossible to meet the associated welding stress requirements.

Therefore, a filling strategy with upper and lower limit functions is adopted. The contour method is used when the layer height exceeds the limit, the equal area method is adopted when the layer height exceeds the limit, and a lifting amount prediction model suitable for the filling layer of large-diameter pipelines is established.

## 3. Establishment of Prediction Model of Uplift of Filling Layer of Large Diameter Pipeline

### 3.1. Estimation of Lifting Layers of Filling Layer of Large Diameter Pipeline

Estimating the number of uplift layers required for large-diameter pipe filling layers using the contour method. The range value of the number of filling layers n can be derived because the lifting amount hi has a positive effect in the range of 2.5 mm~3.0 mm. Set the current remaining layer height *h* of the filling layer to a fixed value (the current remaining layer height *h* reflects the distance between the current filling surface and the pipe’s outer diameter in mm) and fulfill the following relationship:

(9) $$h=\sum_{i=1}^{n}h_{i}$$

Set the maximum number of layers as *n*_ max, there is:

(10) $$n\_\max=\left[\frac{h}{2}\right]$$

Let the minimum number of layers be *n*_ min, there is:

(11) $$n\_\min=\left[\frac{h}{3}\right]$$

Let the number of intermediate layers calculated theoretically be *n*_ middle, there is:

(12) $$n\_middle=\left[\frac{h}{2.5}\right]$$

### 3.2. Calculation Process of Filling Amount of Each Layer of Filling Layer

Now the calculation process of filling layer height is described as follows:

(1) Set the number of filling layers as *n*, and set the initial value *n* = 0; Set the height of each filling layer as H(*i*);
(2) Determine the current remaining theoretical layer height h, and determine *n*_ max, *n*_min, and n_middle, respectively, according to Equations (10)–(12);
(3) N_ middle is substituted into the equal area method to calculate each H(*i*) value under the equal area method;
(4) The judge that *h(i)* is in the range of 2.5 mm~3.0 mm. If yes, record H(*n + i*) = H(*i*), 1 ≤ I ≤ *n*_ middle, and update the number of layers *n = n + n*_middle, output the number of layers *n* and all layer heights H(*i*) (1 *≤ i ≤ n*), then end all calculations and enter end; If not, enter 4);
(5) Judge whether *h*(1) is within the range of 2.5 mm~3.0 mm. If yes, enter (5); If not, enter (7);
(6) Find when 1 < *j < n*_middle + 1, it meets the value *i* of *h(j)* ≥ 2.5 and *h(j + 1)* < 2.5; Record the value j at this time; Record *h(n + I) = h(i)*, 1 *≤ i ≤ j*; Update the value of *n* as *n = n + j*; Since the storey heights of the following floors from *h(j + 1)* to *h (n*_middle) are less than 2.5 mm, calculate the sum of storey heights from *h(j*+1) to *h(n*_middle) as sum, and set the number of remaining floors as *m*, $m=\left[\frac{sum}{2}\right]$;
(7) Find when 1 < *i < n*_middle, When *h(k)* ≥3 and *h(k+1)* <3 are met, record the value k at this time;Record H(*n +i*) =3, 1 *≤ i ≤ k*; Update the value of *n* as *n = n + k*; Update value *h* as h = h-3*k; Cyclic operation, the final calculated number of layers n and height H(*i*) (1 ≤ *i ≤ n*) are the calculated values.

Using the equal-height method, the equal-area method, and the uplift prediction model with saturation effect, the theoretical filling layer height was calculated for the pipe diameter of 1219 mm and the wall thickness of 22 mm, respectively. The results are shown in Table 2.

The error calculation result of the contour method is:

(13) $$\mu_{1}=\frac{\left|16.92-16.9\right|}{16.9}\times 100\%=0.118\%$$

The error calculation result of the equal area method is:

(14) $$\mu_{2}=\frac{\left|16.89-16.9\right|}{16.9}\times 100\%=0.059\%$$

The error calculation result of the prediction model is:

(15) $$\mu =\frac{\left|16.92-16.9\right|}{16.9}\times 100\%=0.118\%$$

Obtained from the calculation error that the lift prediction model with upper and lower limit functions can meet the requirements for use.

As shown in Table 2, the lift amount of each layer of the filling layer is controlled at 2.5 mm~3.0 mm in the lift amount prediction model with upper and lower limit function, which is the optimal model combining the equal height method and the equal area method when considering the weld width of each weld bead of the filling layer. The calculation process is shown in Table 3.

## 4. Welding Test

**Figure 6:**
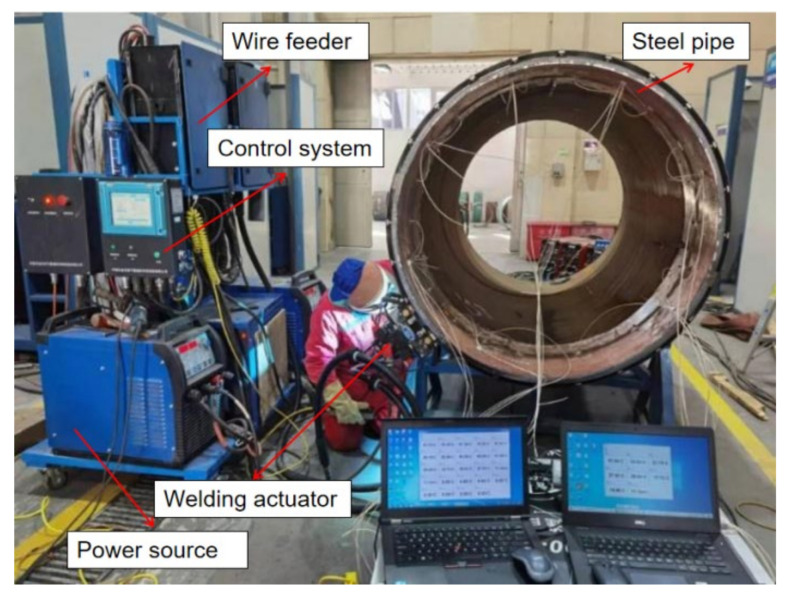
Experiment installation.

**Figure 7:**
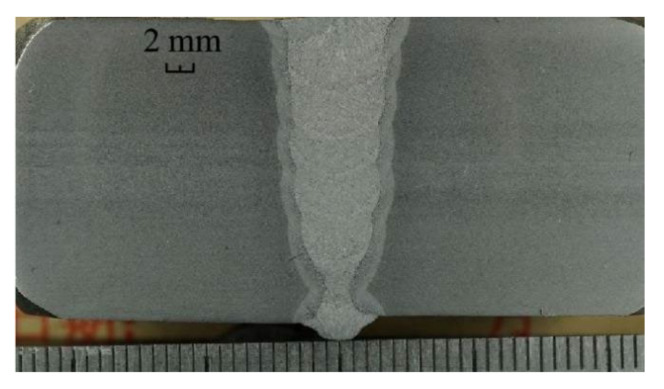
Macro metallographic diagram.

MAG (Metal Active Gas Arc Welding) welding process is adopted. The test material is X80, Ø1219 × 22 mm pipeline steel. The welding material is SG8-P from Austria BOHLER Company. The welding wire has a diameter of Ø1.0 mm. The shielding gas adopts 80%Ar+20%CO_2__._ The metal composition of the test materials and welding consumables is shown in Table 4 and Table 5. The test equipment used the CPP900-W2N automatic welding machine of the China Petroleum and Natural Gas Pipeline Research Institute. The welding power source adopts the Pulse MIG-500 (DC) model and transfers the motion parameters and welding parameters of the welding actuator during the welding process. The wire feeding system provides stable and continuous wire feeding during the welding process. The experiment installation is shown in Figure 6. The welding process parameters during the test are shown in Table 6. Through the test, the macroscopic metallography of the weld is shown in Figure 7.

**Figure 8:**
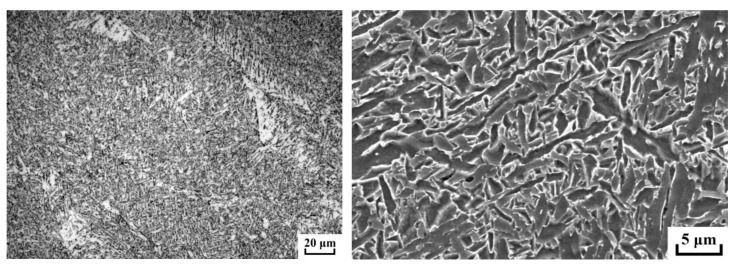
Microstructure of weld metal under SEM.

The main structure of the weld is fine acicular ferrite, and MA islands are distributed on the grain boundary in the form of thin strips, as shown in Figure 8. Acicular ferrite has good strength and toughness, which can improve the mechanical properties of welded joints.

## 5. Conclusions

By studying the path planning of multi-layer multi-pass welding of composite narrow gap grooves of large-diameter oil and gas pipelines, according to the characteristics of composite narrow gap grooves, through the analysis and planning of the filling welding layer and weld bead of narrow gap grooves, multi-layer multi-pass welding of pipelines is analyzed, and a welding strategy that can be used for welding robots outside pipelines is proposed. A prediction model for the lift amount of the welded filler layer outside the pipeline is constructed for the large-diameter composite narrow-gap groove. The welding process parameters for the welded filler layer of the steel pipe with a diameter of 1219 mm and a wall thickness of 22 mm were set using the prediction model. After the workshop welding test, according to the lift prediction model and six times filling welding tests, the lift prediction model can meet the welding requirements of the narrow gap groove of composite materials. The filled weld is flush with the edge of the groove, which is the subsequent cover welding process laid a good foundation. The lift force prediction model is beneficial to scholars who study welding path planning for multi-layer multi-pass pipelines. It lays a foundation for the determination of pipeline welding process parameters and offline welding process programming.

## Figures and Tables

**Table 1:** Layer height of theoretical filling layer with a pipe diameter of 1219 mm.

Filler Layer Theory of the Height *h_i_* Methods	Equal Height Method (mm)	Equal Area Method (mm)
1	2.82	3.44
2	2.82	3.11
3	2.82	2.85
4	2.82	2.65
5	2.82	2.49
6	2.82	2.35
Calculated total layer height h	16.92	16.89

**Table 2:** The theoretical fill layer height of the three calculation methods.

Filler Layer Theory of the Height *h_i_* Methods	Equal Height Method (mm)	Equal Area Method (mm)	A Lift Prediction Model with Upper and Lower Limits (mm)
1	2.82	3.44	3.00
2	2.82	3.11	3.00
3	2.82	2.85	3.00
4	2.82	2.65	2.80
5	2.82	2.49	2.62
6	2.82	2.35	2.50
Total layer height	16.92	16.89	16.92

**Table 3:** The calculation process of an uplift prediction model with upper and lower limit effect.

Filler Layer Theory of the Height *h_i_* Correction Calculation	1st Calculation (mm)	1st Layer Height Correction Value (mm)	2nd Calculation (mm)	2nd Layer Height Correction Value (mm)	3rd Calculation (mm)	Final Result (mm)
*h*	16.9		10.9		7.9	16.9
n_middle	6		4		3	6
1st layer	3.44	3.00				3.00
2nd layer	3.11	3.00				3.00
3rd layer	2.85		3.02	3.00		3.00
4th layer	2.65		2.80		2.80	2.80
5th layer	2.49		2.62		2.62	2.62
6th layer	2.35		2.47		2.48	2.50
Total layer height						16.92

**Table 4:** Chemical composition of base metal (wt%) [26].

Material	C	Si	Mn	P	S	Mo	Ni+Cr+Cu	Nb+V+Ti	Ceq
X80	0.05~0.07	0.25	≤1.80	0.01	0.001	≤0.35	≤0.50	≤0.15	0.42~0.44

**Table 5:** Chemical composition of filler metal [1].

Element	C	Si	Mn	P	S	Cr	Mo	Ni	V	Cu	Ti	Al
Filler metal	0.05	0.69	1.53	0.004	0.006	0.020	0.004	0.89	<0.001	0.110	0.060	0.003

**Table 6:** Welding process parameters for each layer.

Layers	Welding Parameters	3 O’clock	4 O’clock	5 O’clock	6 O’clock
1st layer	Welding speed (cm/min)	59.9	55.1	55.5	52.1
	Current (A)	219.2	196.7	210.4	194.4
	Voltage (V)	25.2	25.1	24.7	24.8
	Swing width (mm)	0	0	0	0
2nd layer	Welding speed (cm/min)	45.5	42.8	41.8	38.7
	Current (A)	207.9	198.4	188.2	185.8
	Voltage (V)	25.7	25.2	24.8	23.8
	Swing width (mm)	1.2	1.3	1.7	1.7
	Swing time (ms)	120	120	120	120
	Swing speed (mm/s)	10	10.8	14.2	14.2
	Edge dwell time (ms)	40	60	80	80
3rd layer	Welding speed (cm/min)	45.3	42.5	38.9	39
	Current (A)	193.1	194.1	180.9	176.5
	Voltage (V)	25.5	25.2	23.8	24.5
	Swing width (mm)	1.2	1.3	1.7	1.7
	Swing time (ms)	120	120	120	120
	Swing speed (mm/s)	10	10.8	14.2	14.2
	Edge dwell time (ms)	40	60	80	80
4th layer	Welding speed (cm/min)	47.7	43.8	37.7	37.1
	Current (A)	203	202.7	187.1	176
	Voltage (V)	25.3	24.4	23.8	24
	Swing width (mm)	1.8	2	2.4	2.4
	Swing time (ms)	120	120	120	120
	Swing speed (mm/s)	15	16.7	20	20
	Edge dwell time (ms)	40	60	80	80
5th layer	Welding speed (cm/min)	47.3	43.6	41.7	36.9
	Current (A)	199.2	184	177.5	189.1
	Voltage (V)	25.3	24.8	24.3	23.8
	Swing width (mm)	1.8	2	2.4	2.4
	Swing time (ms)	120	120	120	120
	Swing speed (mm/s)	15	16.7	20	20
	Edge dwell time (ms)	40	60	80	80
6th layer	Welding speed (cm/min)	44	43.5	43.9	41.6
	Current (A)	230	229.4	200.3	170.8
	Voltage (V)	25.4	25.2	24.9	24
	Swing width (mm)	2.4	2.6	3	3
	Swing time (ms)	150	150	150	150
	Swing speed (mm/s)	16	17.3	20	22
	Edge dwell time (ms)	40	60	80	80

## Data Availability

Data available in a publicly accessible repository.
